# Fall Risk Management in Nursing Homes: A Scoping Review

**DOI:** 10.3390/healthcare13243233

**Published:** 2025-12-10

**Authors:** Cecília Gaspar, Larissa Chaves Pedreira, Neuza Reis, Andreia Costa, Ricardo Oliveira Ferreira, Maria Adriana Henriques, Cristina Lavareda Baixinho

**Affiliations:** 1 Nursing Research Innovation and Development Centre of Lisbon (CIDNUR), School of Nursing, University of Lisbon, 1600-096 Lisbon, Portugal; cecilia.gaspar@campus.esel.pt (C.G.); nreis@esel.pt (N.R.); andreia.costa@esel.pt (A.C.); rf@esel.pt (R.O.F.); ahenriques@esel.pt (M.A.H.); 2 Santa Casa da Misericórdia de Lisboa, 1200-470 Lisbon, Portugal; 3 School of Nursing, Universidade Federal da Bahia, Salvador 40110-907, Brazil; larissa.pedreira@uol.com.br; 4 Instituto de Saúde Ambiental, Faculdade de Medicina, University of Lisbon, 1649-028 Lisbon, Portugal; 5 Laboratório Associado TERRA, University of Lisbon, 1349-017 Lisbon, Portugal; 6 Center for Innovative Care and Health Technology (ciTechcare), 2414-016 Leiria, Portugal

**Keywords:** fall, older adult, prevention/intervention, nursing homes, rehabilitation

## Abstract

**Background**: Population aging represents a growing public health challenge, with falls being one of the leading causes of morbidity, mortality, and loss of autonomy among older adults. In nursing homes, the risk is exacerbated by physical, cognitive, and environmental factors, yet which interventions are most actionable in nursing homes remains unclear, requiring effective and evidence-based prevention strategies. **Objective**: We aimed to map the evidence on interventions in fall risk management among institutionalized older adults, highlighting assessment, exercise, environmental, and educational strategies. **Methods**: A scoping review was conducted according to JBI guidelines. The search was carried out in MEDLINE, CINAHL, Scopus, Cochrane, and Web of Science. The review included studies, published between 2019 and 2024, involving older adults (≥65 years) in nursing homes, focusing on nursing interventions for fall risk management. **Results**: The initial search identified 1146 records across electronic databases and gray literature sources. After removing duplicates and screening titles, abstracts, and full texts, 18 studies met the inclusion criteria and were included in this scoping review. The inclusion criteria were as follows: (i) fall risk assessment, using functional and clinical scales such as the Timed Up and Go (TUG), Berg Balance Scale (BBS), Morse Fall Scale, to identify predisposing factors; (ii) exercise programs, such as the Otago Exercise Program and multicomponent training, which demonstrated benefits in strength, balance, and mobility; (iii) environmental modifications, aimed at reducing extrinsic risks and promoting safer surroundings; and (iv) educational programs, targeting professionals, residents, and families, with positive effects on adherence to preventive practices and on strengthening the safety culture. **Conclusions**: Effective fall risk management in nursing homes requires an integrated, multidisciplinary, and person-centered approach, with nurses playing a central role in assessment, coordination, and implementation of interventions. However, gaps remain regarding standardization, validation of assessment tools specific to the institutional context, and evaluation of long-term outcomes.

## 1. Introduction

The aging of the global population has significant public health implications, particularly regarding falls among older adults. According to the World Health Organization, the proportion of people aged 60 years and over is expected to double from 12% in 2015 to 22% by 2050, reaching approximately 2.1 billion individuals worldwide [1]. This demographic shift is accompanied by an increased prevalence of chronic diseases, frailty, and mobility impairments, all of which contribute to a higher risk of falls. Falls are among the leading causes of morbidity and mortality in older adults, accounting for approximately 684,000 deaths and over 37 million falls requiring medical attention each year globally [2]. Such figures highlight the urgent need for effective strategies to prevent falls and mitigate their consequences for individuals and health systems worldwide.

In institutionalized settings, such as nursing homes, the risk of falls is considerably higher compared to community-dwelling older adults. On average, between 30% and 50% of residents experience at least one fall annually [3], often resulting in fractures, head injuries, and functional decline. This heightened risk is linked to multiple intrinsic and extrinsic factors, including cognitive impairment, reduced mobility, chronic diseases, environmental barriers, and polypharmacy [4]. Additionally, fear of falling (ptophobia) represents a psychological burden that limits daily activity, reduces confidence, and increases the likelihood of subsequent falls [5,6].

Effective fall prevention requires a systematic, multidisciplinary approach. Recent studies emphasize that nursing leadership, staff training, and interprofessional communication are central to ensuring the successful implementation of preventive measures [7,8,9]. A validated framework for fall risk management in nursing homes organizes interventions into three domains: (1) preparing the institution; (2) managing fall risk throughout institutionalization; and (3) leading communication and training [7]. In the first domain, “preparing the institution,” emphasis is placed on establishing organizational policies, allocating adequate resources, and ensuring environmental safety to support a proactive culture of fall prevention. The second domain, “managing fall risk throughout institutionalization,” includes systematic risk assessment, individualized care planning, implementation of exercise and rehabilitation programs, medication review, and monitoring of environmental hazards. The third domain, “leading communication and training,” highlights the importance of continuous staff education, interdisciplinary collaboration, and timely information sharing to ensure consistent application of evidence-based fall prevention strategies.

This framework is relevant to the present review because it highlights the multidimensional nature of fall risk management in institutional settings, encompassing clinical assessment, exercise programs, environmental modifications, and educational interventions. Within all domains, systematic and continuous fall risk assessment plays a pivotal role in identifying residents at higher risk and guiding tailored interventions [7,8].

Despite growing attention to fall prevention, existing reviews have primarily focused on general older populations or specific interventions, rather than on the comprehensive role of nursing-led strategies in institutional contexts. Moreover, few studies have integrated leadership, communication, and training components into the analysis of fall risk management, leaving a gap in understanding how nursing practice can be optimized in these settings. Previous reviews have typically examined isolated components of fall prevention—for example, exercise interventions, multifactorial programs, or environmental adaptations—without synthesizing how these strategies operate collectively within nursing-led frameworks in long-term care. Furthermore, existing reviews seldom address the organizational and leadership dimensions of fall prevention, such as staff education, communication structures, and institutional readiness. As a result, there is limited evidence on how these elements interact and influence the overall effectiveness of fall risk management in nursing homes. This scoping review seeks to fill that gap by providing an integrated overview that encompasses clinical, organizational, and educational nursing interventions.

A preliminary search conducted in MEDLINE and CINAHL identified some systematic reviews addressing isolated or combined interventions for fall prevention; however, no reviews were found that comprehensively covered all dimensions of fall risk management in nursing homes, from assessment to prevention and recurrence management. This gap underscores the need for a broader synthesis of evidence focused on nursing interventions in long-term care environments such as nursing homes. Although this review focuses on institutionalized older adults, the findings are likely relevant to other high-income countries experiencing similar aging trends.

Therefore, this scoping review aims to map the evidence on interventions in fall risk management among institutionalized older adults, highlighting assessment, exercise, environmental, and educational strategies. By mapping current evidence from 2019 to 2024—a period marked by updated international guidelines and growing global attention to patient safety in long-term care—this review seeks to contribute to an evidence-based framework for improving health outcomes and quality of life for institutionalized older adults. Guided by the PCC (Population, Concept, and Context) framework, this review addresses the following research question: “Which nursing interventions are effective in managing fall risk in nursing homes?”

## 2. Materials and Methods

### 2.1. Study Design

The present scoping review aimed to map and synthesize the scientific evidence on interventions for fall risk management in older adults residing in nursing homes. Considering the current state of knowledge and the exploratory nature of the topic, a scoping review was conducted following the methodological recommendations of the Joanna Briggs Institute (JBI) [9] and in accordance with PRISMA-ScR guidelines [10]. The protocol of this scoping review is registered on the Open Science Framework (OSF): DOI 10.17605/OSF.IO/M5HP4. Studies published from 2019 to 2024 were included in this review. This period was chosen to ensure the incorporation of the most up-to-date and relevant evidence, reflecting current clinical practices, recent international guidelines, and contemporary perspectives on fall-risk management among institutionalized older adults.

The period also encompasses significant developments in patient safety policies and nursing leadership. Earlier studies were excluded because several reviews had already synthesized evidence prior to 2019, and the objective of this scoping review was to map emerging evidence and innovations in nursing interventions during the most recent years.

The choice of a scoping review was motivated by the need to capture the full breadth of nursing-led fall risk management interventions in nursing homes. While systematic reviews provide focused analyses on specific interventions or outcomes, they do not comprehensively address the multidimensional and integrated nature of nursing interventions, including risk assessment, prevention strategies and educational or organizational components. Hence, a scoping review was deemed appropriate to map the existing evidence, identify knowledge gaps, and highlight emerging innovations in nursing practice.

The initial search conducted in MEDLINE and CINAHL identified some systematic reviews addressing isolated or combined interventions for fall prevention but did not reveal comprehensive reviews encompassing all dimensions of fall risk management in nursing homes, from assessment to interventions and recurrence management.

### 2.2. Eligibility Criteria

The research question of this study, guided by the PCC (Population, Concept, and Context) framework, was: “Which nursing interventions are effective in managing fall risk in nursing homes?”

Table 1 presents the inclusion and exclusion criteria. The review included studies involving older adults (≥65 years) in nursing homes, focusing on nursing interventions for fall risk management. Studies including individuals under 65 years of age, interventions unrelated to falls, or settings other than nursing homes were excluded. Studies were also excluded if they were editorials, commentaries, study protocols without results, or lacked sufficient methodological details to assess relevance to fall risk interventions. Studies published in Portuguese, English, Spanish, or French with full-text availability were considered eligible. Articles in other languages were excluded. This criterion ensured feasibility in screening, data extraction, and accurate interpretation of study findings.

### 2.3. Searching and Selection of Studies

The literature search was conducted in March 2024 and updated in April 2025, across five electronic databases: MEDLINE (via PubMed), CINAHL (via EBSCOhost), Scopus, Cochrane Library, and Web of Science. The search strategy was adapted for each database, combining controlled vocabulary (such as MeSH terms) with free-text keywords.

Search terms were selected based on prior literature on falls in older adults, key concepts in nursing interventions, and consultation with a research librarian. Synonyms and controlled vocabulary were included to ensure comprehensive coverage of relevant studies.

The main search terms included: “fall prevention,” “falls in older adults,” “fall risk,” “falls in nursing homes,” “nursing interventions,” “nurse-led interventions,” “rehabilitation,” “nursing care,” “older adults,” and “nursing home.” The descriptors were adapted for each database. The search strategy used in MEDLINE and CINAHL are presented in Appendix A.

Grey literature sources were also searched to capture studies not indexed in electronic databases, including Google Scholar and the Portuguese Open Access Scientific Repository. Documents such as governmental reports, dissertations, conference proceedings, e-books, manuals, and guidelines were included if they provided empirical data or detailed descriptions of nursing interventions relevant to fall risk management.

To facilitate the organization and selection of articles after the search process, the Rayyan^®^ platform was used. Screening and data extraction were carried out independently by two researchers (C.G. and C.L.B.). When consensus could not be reached, a third researcher (L.P.) was consulted to make the decision. Documents obtained through the gray literature search that could not be uploaded into the software were digitized and analyzed independently by the two reviewers.

### 2.4. Quality Assessment and Data Extraction

Although methodological quality assessment is not mandatory in scoping reviews [9], all included studies were evaluated for their relevance and appropriateness to the research question. A standardized data extraction form was developed to collect information on study characteristics, population details, intervention components, outcomes related to fall risk, and implementation factors. Data extraction was conducted independently by two reviewers (C.G. and C.L.B.) and supervised by a third reviewer (L.P.) in cases of discrepancies.

### 2.5. Data Synthesis

Due to the heterogeneity of study designs, interventions, and populations, a narrative synthesis was undertaken. Extracted data were charted in a matrix to facilitate comparison across studies, identify patterns, highlight promising interventions, and detect gaps in knowledge regarding nursing-led fall management in nursing homes.

### 2.6. Quality Appraisal

In accordance with JBI guidance for scoping reviews, no formal risk-of-bias assessment was conducted. However, to ensure methodological transparency, a brief appraisal of the relevance and methodological appropriateness of included studies was performed using five key items adapted from JBI critical appraisal tools. These items assessed: (1) clarity of participant characteristics, (2) adequacy of intervention description, (3) appropriateness of outcome measures, (4) consistency between objectives and conclusions, and (5) relevance to the review question. This appraisal was used exclusively to support interpretive context and not to exclude studies.

### 2.7. Ethical Considerations

As this is a literature review, institutional ethical approval was not required. All included studies reported ethical approval from their respective committees, as stated in the original publications. The data supporting the conclusions of this review, including the complete list of included studies and data extraction forms, are available upon request. There are no restrictions on the availability of Supplementary Materials or information.

## 3. Results

**Figure 1:**
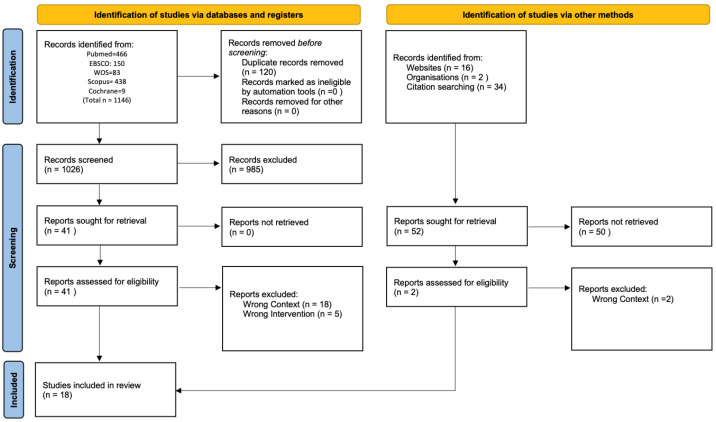
Flow diagram of study selection.

The initial search yielded 1146 records identified across various electronic databases: PubMed (466), EBSCO (150), Web of Science (83), Scopus (438), and Cochrane (9). After removing duplicates, 1026 records were screened based on titles and abstracts. Of these, 41 studies were selected for more detailed analysis following full title and abstract review. The gray literature search identified 16 documents from websites and 2 from organizational sites. From the included articles, 34 additional references were identified for screening. After full-text assessment, 18 studies met the inclusion criteria and were included in the final review[11,12,13,14,15,16,17,18,19,20,21,22,23,24,25,26,27,28] (Figure 1).

### 3.1. Characteristics of Included Studies

In terms of study types, primary studies, secondary studies, and guidelines specific to fall risk management were included. Reviews were included to provide a broader synthesis of evidence, capturing interventions that might not be fully reported in individual studies and offering a comprehensive overview of nursing-led strategies in fall risk management. This approach also allowed identification of emerging interventions, trends, and knowledge gaps that may not be apparent when only consulting original studies, ensuring a more complete mapping of current evidence.

The analysis of the studies reveals a wide geographic distribution, with research conducted in various international contexts specifically in Austria [11,22], Sweden [12], Spain [14], Belgium [17,18,20], Ireland [15,16], Hungary [21], Poland [13], and Portugal [26]—reflecting the growing concern in European countries regarding fall management strategies in institutional settings such as nursing homes. Contributions from North America were also noted, with studies from Canada [23,24,27], highlighting multifactorial interventions and quality improvement projects in residential care facilities for older adults. Asia was represented by studies conducted in China [19,25] and Turkey [28], demonstrating tailored approaches adapted to older populations with diverse clinical and cultural profiles. This wide variety of contexts enhances the external validity of the findings, offering a more comprehensive understanding of practices and challenges associated with fall risk management, and underscoring the global relevance of fall prevention strategies in nursing homes.

### 3.2. Interventions for Fall Risk Management

Fall risk assessment was reported in 16 studies [11,12,13,14,16,17,18,19,20,21,22,23,24,25,26,27] and is recognized as a fundamental step for early identification of predisposing factors. Assessment methods include validated scales, clinical observations, and multidimensional risk analysis encompassing physical, cognitive, environmental, and pharmacological aspects. Key instruments include:

- Timed Up & Go (TUG) [14,17,18,19,21,25,26]—mobility assessment; predictive accuracy varied (sensitivity 29–50%, specificity 83–96%) [17,18,27].
- Berg Balance Scale (BBS) [19,26,28]—balance; MD = +5.55 points (OEP study, *p* < 0.001);
- Toulouse Saint Louis University Mini Falls Assessment (TSLUMFA) [20]—cut-off ≤ 21 indicated high risk; predictive validity confirmed.
- 30-Second Chair Stand Test (30 s-CST) [19,25,28]—lower limb strength; OEP showed MD = +4.32 repetitions, *p* < 0.001.

Exercise programs were implemented in 11 studies [11,12,13,14,16,19,21,22,23,25,28], aiming to improve mobility, muscle strength, and balance. Programs varied from multicomponent training [14,21,23] to progressive resistance training using inertial devices [13], including sensor-assisted interventions [17,18].

- Otago Exercise Program (OEP): strength, balance, and mobility training, 2–3×/week, 12–16 weeks [19,25,28]. Effect sizes: fall risk reduction MD = −0.84, *p* < 0.00001; BBS +5.55 points, TUG −6.39 s; 30 s-CST +4.32 repetitions [19].
- Multicomponent training: balance + strength + aerobic + functional exercises; 12-week program improved SPPB (*p* = 0.003) and showed positive trends in TUG, Functional Reach Test, 6MWT, though changes in fall rates were non-significant [21].
- Dual-task training: cognitive + physical exercises improved cognitive performance but did not significantly reduce falls [14].
- Progressive resistance training with inertial devices: improved strength by 37–69%, balance (+29% Tinetti), and gait speed (+12.8%) [13].

Environmental modifications were addressed in 5 studies [11,12,16,23,24] and included furniture adjustments, use of protective devices, improved lighting, and removal of obstacles. Reduction in environmental hazards contributed to fewer falls in combination with exercise and education. Specific RCT evidence for environmental-only interventions was limited [11,16,24].

Educational programs were highlighted in 7 studies [11,15,16,23,24,25,28], emphasizing staff training, resident education, and involvement of families. Structured and ongoing training, combined with practical materials, enhances awareness, knowledge, and adherence to preventive measures. Staff training consistently improved knowledge and adherence to fall prevention strategies; improvements in fall rates were small but positive [11,15,24].

RCTs with multicomponent programs showed significant functional improvements and non-significant but positive trends in fall reduction [21,23,25]. Only 6 studies focused on a single intervention domain [15,17,18,20,26,27], supporting the effectiveness of integrated approaches, particularly when multidisciplinary teams are involved.

Table 2 presents a summary of the included studies, including study type, intervention domains, and key outcomes. Detailed information on each study, including full interventions and results, is available in Supplementary File S1.

## 4. Discussion

This study provides important insights into the multidimensional interventions implemented by rehabilitation specialist nurses for fall management in nursing homes, highlighting the role of assessment, exercise, environmental adaptations, and education in reducing fall risk. Although two studies [29,30] were excluded from the final synthesis due to age criteria, they are discussed here exclusively as contextual evidence to support the interpretation of the findings. These studies were not part of the analysed dataset but are included because their results reinforce the relevance of integrated, personalized fall-prevention strategies. These findings complement the present results and reinforce the value of multidimensional approaches in nursing home settings. The results of this study emphasize the central role of fall risk assessment in guiding preventive interventions. Instruments such as the Timed Up & Go Test (TUGT) and the Toulouse Saint Louis University Mini Falls Assessment (TSLUMFA) can support the early identification of residents at higher risk of falls. However, evidence [31] indicates that the Timed Up and Go test alone has limited predictive accuracy in community-dwelling older adults, with low sensitivity and moderate specificity. Therefore, the TUGT should be used cautiously and always in combination with other clinical assessments and multifactorial risk factors [17,18,20,31]. Combining formal assessments with questions about previous falls represents a practical, low-cost strategy widely recommended in institutional settings [6,8,28,32]. To increase the utility of assessment tools in nursing-home practice, key instruments can be interpreted using indicative thresholds to guide intervention planning:

- Timed Up & Go (TUG): ≥13.5 s indicates high fall risk, suggesting the need for targeted balance and mobility interventions, possibly supervised exercise programs.
- 30-Second Chair Stand Test (30 s-CST): <12 repetitions signals lower-limb weakness, indicating the need for progressive resistance training and functional strengthening.
- Berg Balance Scale (BBS): ≤45 points reflects clinically significant balance impairment, guiding clinicians to implement multicomponent balance and strength programs.
- Toulouse Saint Louis University Mini Falls Assessment (TSLUMFA): ≤21 indicates high fall risk, supporting comprehensive, individualized preventive strategies.
- Short Physical Performance Battery (SPPB): ≤9 suggests moderate-to-severe functional limitation, prompting intensive, multidisciplinary interventions.

Based on current evidence and institutional guidelines, re-evaluation of fall risk is recommended at least every three months, or earlier following any significant health change, fall event, or medication adjustment. This periodic reassessment ensures the timely adaptation of preventive strategies and individualized care plans. Regarding exercise programs, evidence from this study aligns with previous research supporting the Otago Exercise Program (OEP) in reducing fall risk through improvements in balance, muscle strength, and functional mobility [19,25,28,32]. Additionally, although multicomponent exercise programs combining balance, strength, and aerobic training have shown promising functional improvements, the overall evidence remains limited and heterogeneous. The suggestion that combined resistance and aerobic training may offer additive or superior benefits is largely supported by small randomized controlled trials (typically with samples between 20 and 80 participants) and a few meta-analyses with low-to-moderate GRADE certainty due to methodological variability, imprecision, and risk of bias. Importantly, robust evidence specifically involving nursing-home residents—particularly those with cognitive impairment—is scarce, meaning that the potential superiority of combined training remains preliminary and should be interpreted cautiously; further robust trials are needed to confirm these effects [21,22]. The OEP has been effectively adapted for institutionalized older adults. The recommended frequency is three sessions per week, with each session lasting approximately 30 to 45 min, over a minimum period of six months. Evidence suggests that benefits in balance and lower limb strength become more evident after 12 weeks, but sustained implementation for ≥6 months is necessary to achieve significant reductions in fall incidence and to maintain functional gains [19,25,28,32]. Environmental modifications, such as handrails, obstacle removal, improved lighting, and safe furniture arrangements, have demonstrated effectiveness, particularly when combined with exercise and educational programs [7,16,23,33]. These interventions reduce extrinsic risk factors and create safer environments that complement other preventive strategies. Educational interventions for both staff and residents are also crucial. Training caregivers, raising awareness of fall risks, and promoting the correct use of assistive devices contribute to fall reduction [18,20]. Such strategies empower professionals and residents, supporting adherence to preventive measures and fostering a culture of safety [34]. Overall, the findings underscore the importance of a multifactorial approach, integrating clinical assessment, exercise, environmental adaptation, and education [6,7,8,19,27]. Tailoring interventions to individual resident characteristics—including frailty, cognition, and fall history—is recommended. For example, residents with a Clinical Frailty Scale score ≥ 5, Mini-Mental State Examination < 24, or history of ≥1 fall in the past year may benefit from more intensive, supervised interventions. Limitations of the current study include the short follow-up period. Evidence suggests that follow-up durations of ≥6 months are advisable to adequately evaluate the sustainability of intervention effects [22,32]. The heterogeneity of intervention designs, participant characteristics, and outcome measures also limits comparability. Additionally, the validation of fall risk assessment tools specifically for institutionalized older adults remains a research priority, as existing instruments may lack sensitivity or specificity in this population [20,26,32].

In conclusion, fall risk management in nursing homes requires an integrated, multidisciplinary, and personalized approach that considers both intrinsic and extrinsic risk factors. Combining precise assessment tools, evidence-based exercise programs (including potentially combined resistance and aerobic training), environmental adaptations, and targeted educational strategies represents the most comprehensive and effective approach. Follow-up periods of ≥6 months are recommended to monitor outcomes and ensure sustainability of interventions [6,32]. Regular fall risk reassessment (every three months or after major health changes) and continuous exercise implementation (three 30–45 min OEP sessions per week for ≥6 months) are recommended to ensure sustained benefits in balance, mobility, and fall reduction.

This systematic review has certain limitations arising from methodological choices, such as language restrictions and the requirement for free full-text availability, which likely led to the exclusion of some studies relevant to the research question. Other limitation is the restriction of the search to studies published between 2019 and 2024. Although this time frame was chosen to capture the most recent and relevant evidence, it may have excluded earlier research that could offer valuable insights into long-term trends or foundational concepts related to fall-risk management in institutionalized older adults. As a result, the findings of this review should be interpreted with consideration of this temporal constraint. It is also important to note that the included studies are heterogeneous in terms of design, participants, and type of intervention. Another limitation is that, in general, systematic reviews do not assess the methodological quality of the included studies or the potential for bias [23].

One of the strengths of this study is the large sample that was obtained through the search method used, which provided a wide range of relevant information on the subject under study.

## 5. Conclusions

The analysis of the 18 studies included in this scoping review revealed that effective fall management depends on a combination of strategies, such as adapted physical exercise programs (notably the Otago Exercise Program and structured multicomponent exercises), environmental modifications, and educational programs targeted at professionals, residents, and families.

The results reinforce the central role of nurses in the systematic assessment of fall risk, the planning of individualized interventions, and the coordination of educational and preventive actions in collaboration with the multidisciplinary team. Moreover, the implementation of evidence-based practices proved crucial for improving balance, strength, mobility, and, most importantly, for reducing the incidence of falls and their consequences.

Future research should focus on longitudinal studies with follow-up durations of at least 6–12 months to evaluate the sustainability of intervention effects, particularly for exercise, educational, and environmental strategies. Studies should also aim to validate sensitive, context-specific fall risk assessment tools for institutionalized older adults.

Practical recommendations include integrating emerging technologies, such as wearable sensors or telemonitoring, with ongoing staff training and regular environmental audits. Implementing structured protocols for exercise progression, fall risk reassessment, and caregiver education can enhance safety, independence, and quality of life for residents in nursing homes.

## Figures and Tables

**Table 1:** Eligibility Criteria. Lisbon, 2025.

	Inclusion Criteria	Exclusion Criteria
Population	Older adults (≥65 years)	Individuals under 65 years
Concept	Nursing interventions focused on fall risk management (risk evaluation, prevention of falls, interventions, care after falls, control of fear of falling)	Interventions not related to falls
Context	Studies conducted in nursing homes	Studies conducted in the community, hospitals, home care, or rehabilitation units

**Table 2:** Summary of Included Studies and Nursing Interventions for Fall Risk Management (Condensed). Lisbon, 2025.

Article	Country	Study Type	Intervention Domains	Key Outcomes
[11]	Austria	Systematic Review	Assessment, Exercise, Environmental, Education	Assessment recommended for all; exercise limited for frail residents; staff training strongly recommended
[12]	Sweden	RCT	Assessment, Exercise, Environmental	High-Intensity Functional Exercise (HIFE) program improved balance; no significant fall reduction; multifactorial adjustments suggested
[13]	Poland	RCT	Progressive Resistance Training with inertial devices	Increased strength & balance; safe and feasible; reduced fall risk
[14]	Spain	Secondary RCT	Exercise	Multicomponent training reduced falls; dual-task cognitive training less effective
[15]	Ireland	Systematic Review	Education	Staff training key; lack of standardization limits effectiveness
[16]	Ireland	Cross-Sectional	Assessment, Exercise, Environmental, Education	Environmental modifications prioritized; training gaps; need for proactive risk assessment
[17]	Belgium	Longitudinal Observational	Assessment	Timed Up & Go Test (TUGT) with sensors improved fall prediction; low false positives
[18]	Belgium	Longitudinal Observational	Assessment	Timed Up & Go Test (TUGT) + 6-Minute Walk Test (6MWT) with AI increased predictive accuracy
[19]	China	Systematic Review & Meta-Analysis	Assessment, Exercise	Otago Exercise Program (OEP) improved strength, balance, mobility; fall risk decreased
[20]	Belgium	Prospective	Assessment	Toulouse Saint Louis University Mini Falls Assessment (TSLUMFA) validated; cut-off ≤21 indicated high risk
[21]	Hungary	RCT	Assessment, Exercise	12-week multicomponent program improved physical function; fall trends positive but non-significant
[22]	Austria	Systematic Review & Meta-Analysis	Exercise	Balance and tech-assisted exercises reduced falls; frail residents showed increased risk
[23]	Canada	Systematic Review & Meta-Analysis	Assessment, Exercise, Environmental, Education	Multifactorial interventions reduced falls; exercise effectiveness in dementia uncertain
[24]	Canada	Quality Improvement	Assessment, Environmental, Education	Individualized plans reduced fall rate and fall-related injuries
[25]	China	Quasi-Experimental	Assessment, Exercise, Education	Group-based Otago Exercise Program (OEP) improved mobility, strength, reduced fear of falling
[26]	Portugal	Integrative Review	Assessment	Functional tests and scales used; combined tools may be impractical; early assessment essential
[27]	Canada	Retrospective Cohort	Assessment	InterRAI Clinical Assessment Protocol (interRAI CAP) best predictive accuracy; inclusion of diagnoses improved prediction
[28]	Turkey	RCT	Exercise, Education	Otago Exercise Program (OEP) reduced falls, improved Berg Balance Scale (BBS) (+6) and 30 s chair stand (+4 repetitions)

Legend: Intervention domains include assessment (clinical, functional, and pharmacological evaluation), exercise (physical training, multicomponent programs, OEP), environmental modifications (space adaptation, protective devices), and educational programs (staff/resident training).

## Data Availability

The original contributions presented in the study are included in the article/Supplementary Materials, further inquiries can be directed to the corresponding author.

## Supplementary Materials

The following supporting information can be downloaded at: https://www.mdpi.com/article/10.3390/healthcare13243233/s1.

## Appendix A

**Table A1:** Search Strategy. Lisbon, 2025.

Base	Search Strategy
Medline	S1—(Age*[Title/Abstract]) OR (Elderl*[Title/Abstract])) OR (older person[Title/Abstract])) OR (older people[Title/Abstract])) OR (older population[Title/Abstract])) OR (geriatric[Title/Abstract])) OR (aged[MeSH Terms])) OR (adult, frail older[MeSH Terms])) OR (adults, frail older[MeSH Terms])) OR (frail older adult[MeSH Terms])) OR (frail older adults[MeSH Terms])) OR (older adult, frail[MeSH Terms])) OR (elderly[MeSH Terms])) OR (elderly, frail[MeSH Terms])) OR (elderly, functionally impaired[MeSH Terms]) S2—(accidental fall[Title/Abstract]) OR (accidental falls[Title/Abstract])) OR (fall*[Title/Abstract])) OR (risk of fal*[Title/Abstract])) OR (fall prevention[Title/Abstract])) OR (fall intervent*[Title/Abstract])) OR (Risk assessment[Title/Abstract])) OR (prevention programs[Title/Abstract])) OR (rehabilitation programs[Title/Abstract])) OR (physical exercise[Title/Abstract])) OR (psychoeducational interventions[Title/Abstract])) OR (Activities of Daily Living training[Title/Abstract])) OR (accidental fall[MeSH Terms])) OR (accidental falls[MeSH Terms])) OR (accident prevention[MeSH Terms])) OR (accident preventions[MeSH Terms])) OR (assessment, risk benefit[MeSH Terms])) OR (activity, physical[MeSH Terms])) OR (activities, physical[MeSH Terms])) OR (rehabilitation nursing[MeSH Terms])) OR (rehabilitation nursings[MeSH Terms]) S3—(“nursing home”[Title/Abstract] OR “nursing homes”[MeSH Terms] OR “nursing homes”[MeSH Terms] OR “homes for the aged”[MeSH Terms] OR “nursing homes”[MeSH Terms]) S1 and S2 and S3
CINAHL	S1—(Age*[Title/Abstract]) OR (Elderl*[Title/Abstract])) OR (older person[Title/Abstract])) OR (older people[Title/Abstract])) OR (older population[Title/Abstract])) OR (geriatric[Title/Abstract])) OR (Aged, 80 and Over[Title/Abstract])) OR (adult, frail older[Title/Abstract])) OR (adults, frail older[Title/Abstract])) OR (frail older adult[Title/Abstract])) OR (frail older adults[Title/Abstract])) OR (older adult, frail[Title/Abstract])) OR (elderly, frail[Title/Abstract])) S2—(accidental fall*[Title/Abstract]) OR (accident prevention[Title/Abstract])) (fall*[Title/Abstract])) OR (risk of fal*[Title/Abstract])) OR (fall prevention[Title/Abstract])) OR (fall intervent*[Title/Abstract])) OR (Risk assessment[Title/Abstract])) OR (Fall Risk[Title/Abstract])) OR (Risk management[Title/Abstract])) OR (prevention programs[Title/Abstract])) OR (rehabilitation programs[Title/Abstract])) OR (physical exercise[Title/Abstract])) OR (exercise[Title/Abstract])) OR (psychoeducational interventions[Title/Abstract])) OR (psychoeducational [Title/Abstract])) OR (Activities of Daily Living training[Title/Abstract])) OR (Activities of Daily Living [Title/Abstract])) OR (rehabilitation nurs*[Title/Abstract])) S3—(“nursing home”[Title/Abstract] OR “homes for the aged”[Title/Abstract] S1 and S2 and S3
