# Supplementary material for: Fall Risk Management in Nursing Homes: A Scoping Review
Source: Healthcare (Basel). 2025 Dec 10;13(24):3233. doi: 10.3390/healthcare13243233 (PMC12733304; doi:10.3390/healthcare13243233)
Supplement: Supplementary file 1 [file healthcare-13-03233-s001.zip › healthcare-3971735-supplementary.pdf]

healthcare MDPI

Supplementary File S1

Article / (Year) ǀ Country	Study Type / Objective / Sample	Intervention Domains	Results
[11] (2022) Áustria	Systematic Literature Review<br><br>To provide nurses with an overview of all relevant research literature on fall prevention and the development of a clinical practice guideline for fall prevention in older adults<br><br>Number of included articles: 79	Assessment, Exercise, Environmental, Education	Fall Risk Assessment: It is recommended to assess all older adults for fall risk. There is no recommendation to use a specific risk assessment tool. Periodic review of medications that may increase fall risk is recommended.<br>Exercise Programs: In nursing homes, physical exercise showed benefits only when excluding frailer residents (weak recommendation). No significant difference was observed between combined exercises and targeted exercises (balance, strengthening). Exercise increased fall risk in frail residents and is therefore not recommended for this group. Tai Chi did not reduce falls but decreased fear of falling; it is recommended only for those with this concern. Interventions using technical devices showed a positive impact and received a strong recommendation despite associated costs.<br>Environmental Modifications: No RCTs specifically addressing environmental adaptations for fall prevention in hospitals and nursing homes were found. Non-RCT studies included environmental adaptations within multifactorial strategies. The use of slippers is a significant risk factor for falls in nursing homes. Low-height beds are not recommended for fall prevention. Alarms and sensors received a weak recommendation due to limited evidence. Hip protectors did not reduce the fall rate but slightly decreased hip fractures.<br>Educational Programs: Older adults without cognitive impairment should receive intensive education on fall risk. Active caregiver training is strongly recommended in residential care facilities. More intensive training programs (group sessions, materials, audits, feedback) showed small benefits compared to less intensive

			training. Active staff training received a strong recommendation due to its positive impact on knowledge and slight reduction in falls.
[12] (2019) Suécia	Randomized Clinical Trial<br>To investigate the effects of exercise on fall prevention in residents with dementia in nursing homes and to determine whether the effects differed according to sex, type of dementia, or balance improvement. Fall-related injuries were also analyzed.<br>Participants: 186 (141 women and 45 men)	Assessment, Exercise, Environmental	Fall Risk Assessment: Differences were observed according to type of dementia: participants with Alzheimer's disease had a higher fall rate during the intervention in the exercise group, but this difference diminished at follow-up. Medication adjustments and review of medical conditions were suggested as part of a multifactorial fall prevention approach.<br><br>Exercise Programs: The HIFE program (High-Intensity Functional Exercise) was conducted 2–3 times per week for 4 months. Exercises focused on muscle strength (squats, leg raises, knee extensions), balance (single-leg stance, weight shifting, walking in a straight line), and mobility (walking with obstacles, ascending/descending stairs). Balance improved in the exercise group, but there was no significant reduction in fall rate at 6 and 12 months. Exercises reduced the severity of fall-related injuries after 12 months. Cognitive training combined with exercise may be effective for fall prevention.<br><br>Environmental Modifications: Environmental adaptation was included as part of the multifactorial approach (no specific details provided). Recommended assistive devices included walkers, grab bars, and non-slip footwear.
[13] (2020) Poland	Randomized Clinical Trial<br><br>To evaluate the impact of inertial training on upper and lower limb strength in older adults residing in nursing homes. Additionally, to measure the effects of this training on independence, balance, and gait quality.<br><br>Participants: 20 physically inactive older adults (6 women, 14 men; mean age: 76.7 years)	Progressive Resistance Training	Fall Risk Assessment: Improvements in balance (Tinetti Balance Test +29%) and gait speed (+12.8%) are functional indicators that can be used to monitor and manage fall risk. The significant increase in muscle strength helps reduce fall risk, establishing inertial training as an effective risk management strategy. The control group showed no improvements, reinforcing the potential effectiveness of inertial training in fall risk reduction.<br><br>Exercise Programs: Inertial training using the Cyklotren device, 2 times per week for 6 weeks,

	Groups: Inertial training (n = 10) vs. Control (n = 10)		focused on upper and lower limbs with progressive loads. Exercises targeted elbow and knee flexors and extensors. Muscle strength increased between 37.1% and 69.1%, with improvements in balance, gait, and gait speed. The training was well tolerated and safe, with no reported injuries. Inertial training is recommended for inclusion in the routine of older adults in nurssing homes.
[14]<br>(2022)<br>Spain	Secondary analysis of a randomized controlled trial<br>To compare the effects of a multicomponent exercise program and a dual-task exercise program on fall rate and fall incidence, as well as on parameters associated with fall risk in older adults living in nursing homes.<br>Population: 85 older adults from 9 different nursing homes.<br>Inclusion criteria: Age ≥ 70 years, Barthel Index score ≥ 50, Mini-Cognitive Examination score ≥ 20 (Spanish version of the Mini-Mental State Examination), ability to stand and walk (with or without assistive devices).	Exercise	Fall Risk Assessment: Monitoring of the number and incidence of falls through medical record documentation, as part of the risk assessment. The Timed Up & Go (TUG) test and handgrip strength were used to assess physical performance, which are indirect indicators for fall risk management. Handgrip strength asymmetry was also evaluated, as it is a factor related to fall risk.<br>Exercise Programs:<br>Multicomponent group: progressive strength and balance training, twice per week for 3 months.<br>Dual-task group: the same training, but with simultaneous cognitive tasks included in part of the exercises.<br>The multicomponent group significantly reduced falls and improved physical performance (improvement in TUG and reduction in grip strength asymmetry).<br>The dual-task group had a higher risk of falls during and after the intervention, despite showing improvements in cognitive performance during the TUG.<br>Conclusion: Multicomponent training was more effective for fall prevention than simultaneous dual-task training.
[15]<br>(2022)<br>Ireland	Systematic Literature Review<br>To synthesize the evidence on implementation strategies used in fall prevention interventions in nursing homes, analyzing their outcomes both in implementation and in fall reduction.	Education	Educational Programs: Staff education and training were the most frequently used implementation strategies (26 out of 31 studies).<br>Common techniques: educational meetings (17 studies), distribution of educational materials (17), and development of educational resources (13). Team capacity-building is essential for the success of fall prevention interventions.

	Number of articles included: 31 studies, corresponding to 27 different interventions.		Identified challenges: insufficient reporting of implementation strategies; lack of clear standards on which strategies are most effective for successful intervention implementation.<br>Conclusion: Staff training is the most commonly used implementation strategy to support fall prevention interventions. However, the lack of standardized reporting makes it difficult to understand the relationship between implementation strategies and intervention effectiveness.
[16]<br>(2023)<br>Ireland	Descriptive Cross-Sectional Study<br>To explore the current practices of nursing home professionals regarding fall prevention and to gather their suggestions for improving the prevention of falls and related injuries<br>Participants: 155 responses across 13 facilities.	Assessment, Exercise, Environmental, Education	Fall Risk Assessment: Staff placed greater emphasis on extrinsic factors (environment, equipment) than on intrinsic factors (individual risk assessment, mobility, cognition). Low recognition of the importance of medication review and vitamin D supplementation as preventive measures. Continuous training is needed, including proactive fall risk assessment and medication management.<br>Exercise Programs: Physical exercise and mobility programs were acknowledged but mentioned by only 10.8% of staff. Physicians recommended the involvement of physiotherapists and occupational therapists.<br>Environmental Modifications: Environmental assessment and modification were considered the main strategy (40.3%), including non-slip flooring, removal of obstacles, improved lighting, handrails, and locking furniture wheels. A safe environment for injury prevention was associated with low beds, cushioned mats, and appropriate furniture (58.7%). Use of alarm and call systems (19.3%) and provision of suitable assistive devices (13.1%) were also identified as important strategies.<br>Educational Programs: Staff training on fall risk factors, with specific sessions for caregivers and regular updates (39.5%). Involvement of residents and families in prevention through education (8.5%). Knowledge gaps regarding evidence-based strategies highlight the need for ongoing institutional training.
[17]<br>(2022)	Longitudinal Observational Study	Assessment	Fall Risk Assessment:

Belgium	To evaluate whether adding an inertial sensor to the Timed Up and Go (TUG) test improves the predictive accuracy of fall risk in nursing home residents.<br>Participants: 73 nursing home residents in Belgium, aged 65 years or older.		Instrument evaluated: Timed Up & Go test with an inertial sensor (i+TUG).<br>Parameters analyzed: Angular velocity, total test time, use of walking aids.<br>i+TUG model performance:<br>• Overall accuracy: 74%<br>• Specificity: 95.9% (excellent for identifying residents who will not fall)<br>• Sensitivity: 29.2% (low for identifying residents who will fall)<br>The i+TUG is a useful tool for ruling out residents at low fall risk, potentially avoiding unnecessary interventions
[18]<br>(2020)<br><br>Belgium	Longitudinal Observational Study<br>To evaluate whether combining the Timed Up and Go (TUG) and Six-Minute Walk Test (6MWT), complemented with inertial sensors, improves the prediction of fall risk in nursing home residents.<br>Population: 73 nursing home residents in Belgium, aged ≥ 65 years.	Assessment	Fall Risk Assessment:<br>Traditional TUG: accuracy of 65.7%<br>TUG+ (TUG + gait variability): accuracy increased to 73.9%<br>Artificial Intelligence model: accuracy of 75%, with a better balance between sensitivity and specificity<br>Distinguishing patterns between fallers and non-fallers:<br>• Longer TUG time (fallers: 23 s vs. non-fallers: 19 s; $p = 0.035$)<br>• Greater anteroposterior acceleration variability ($p = 0.010$)<br>• Lower complexity of gait patterns ($p = 0.044$)<br>Including gait variability and using AI models increases the accuracy of fall risk assessment, enabling earlier detection and more effective targeting of preventive interventions in nursing homes.
[19]<br>(2023)<br>China	Systematic Review and Meta-Analysis<br>To evaluate the effects of the Otago Exercise Program (OEP) on postural control, fall risk, and other functional parameters in older adults residing in nursing homes.<br><br>Number of studies included: 9 studies, with a total of 546 participants (269 in the OEP group and	Assessment, Exercise	Fall Risk Assessment:<br>Instruments used to measure the program's impact included:<br>• Berg Balance Scale (BBS): balance<br>• Timed Up and Go (TUG): short-distance functional mobility<br>• 30-Second Chair Stand Test (30s-CST): lower limb muscle strength<br>• Six-Minute Walk Test (6MWT): long-distance mobility<br>• Fall incidence and frailty level

	277 in the conventional training group).		Exercise Program – Otago Exercise Program (OEP):<br>Strength and balance training: 22 exercises divided into warm-up, strength, and balance training, with functional progression. Regular walking sessions with progressive duration (gait training).<br>Significant results in fall risk reduction with OEP:<br>• Fall risk reduction: MD = −0.84 ($p < 0.00001$)<br>• Improvement in balance (BBS): MD = +5.55 points<br>• Improvement in TUG: MD = −6.39 s<br>• Increased muscle strength (30s-CST): MD = +4.32 repetitions<br>• Reduction in frailty: RR = 0.35 ($p = 0.001$)<br>• No improvements in 6MWT (long-distance mobility)<br>Conclusion: OEP improves strength, balance, and short-distance mobility, reducing fall risk, but has no impact on long-distance mobility, likely due to low aerobic intensity
[20] (2021) Belgium	Prospective Longitudinal Study<br>To evaluate the predictive ability of the Toulouse Saint Louis University Mini Falls Assessment (TSLUMFA) for falls in older adults residing in nursing homes and to determine an optimal cut-off point to identify those at higher fall risk.<br>Population: 93 older adults residing in nursing homes in Belgium.<br>Inclusion criteria: Age ≥ 65 years; Residency in a nursing home; Preserved cognitive function (Mini-Mental State Examination ≥ 24 points)	Assessment	Fall Risk Assessment:<br>The TSLUMFA evaluates six domains (maximum score: 30 points):<br>• Medication use (including vitamin D supplementation)<br>• Blood pressure<br>• Sitting balance<br>• Standing balance and muscle strength<br>• Gait<br>• FRAIL Score (frailty indicator)<br><br>Key results:<br>The TSLUMFA is an effective tool for predicting falls in nursing home residents. It can be used to identify older adults who require targeted preventive measures. Additional comparative studies with other assessment tools are recommended.
[21] (2024)	Randomized Controlled Trial	Assessment, Exercise	Fall Risk Assessment:

Hungary	To evaluate the impact of a 12-week multicomponent exercise program on reducing the number of falls and improving physical function in older adults residing in nursing homes.<br>Participants: 24 older adults (≥ 65 years), randomly assigned to two groups:		Identified risk factors included: female sex, lower muscle strength, reduced balance, and lower cognitive function.<br>Exercise Program:<br>Supervised multicomponent exercise program (2x/week, 45–60 min, moderate intensity). Exercise components:<br>• Balance: tandem stance, single-leg support, walking with changes of direction<br>• Strength: sit-to-stand, free weight exercises<br>• Aerobic: interval walking<br>• Warm-up and cool-down included<br>Results:<br>Non-significant reduction in falls in the intervention group (IG); Significant improvement in SPPB (p = 0.003); Positive trends in other tests (TUG, FRT, SLS, 6MWT); Control group showed decline in most functional indicators;<br>The multicomponent program was feasible, safe, and beneficial for physical function. It may have potential to reduce fall risk, but larger studies with longer follow-up are needed to confirm effects on falls.
[22] (2020) Austria	Systematic Review and Meta-Analysis of Randomized Controlled Trials (RCTs)<br>This study aimed to provide a comprehensive analysis of the effectiveness of exercise interventions for fall prevention in nursing homes, supporting clinical decision-making.<br>Number of articles included: 9	Exercise	Fall Risk Assessment<br>Exercise Programs:<br>• Balance exercises: postural training, single-leg support, center-of-gravity shifts<br>• Technology-assisted exercises: Wii Balance Board, platforms with visual feedback<br>Main results:<br>• Significant reduction in falls with balance exercises (RR = 0.79) and technology-assisted exercises (RR = 0.55)<br>• No significant reduction in residents with cognitive impairment<br>• Increased fall risk in frail residents (RR = 1.17)<br>• Interventions longer than 6 months were more effective (RR = 0.73)<br>• Moderate frequency (less than 3 times per week) was more effective (low-certainty evidence)

[23] (2021) Canada	Systematic Review and Meta-Analysis<br>To determine the effectiveness of fall prevention interventions in nursing homes and to assess the generalizability of these interventions to individuals with cognitive impairment and dementia.<br>Studies included: 36 articles	Assessment, Exercise, Environmental, Education	Single Interventions:<br>Physical exercise including gait, balance, and functional training, resistance and strength training, stretching, and specific programs such as Tai Chi, yoga, whole-body vibration (WBV), and short-stick exercises. Staff education on person-centered care, risks associated with polypharmacy, and safe medication use. Medication review to reduce psychotropic drugs and inappropriate prescriptions. Vitamin D supplementation.<br>Multiple Interventions:<br>Combination of physical exercise with vitamin D supplementation, urinary incontinence management, nutritional therapy, or podiatry interventions.<br>Multifactorial Interventions:<br>Fall risk assessment, physical exercise, staff education, medication review, environmental adaptations (furniture adjustments, use of protective devices and alarms), and podiatry interventions.<br>Key Findings:<br>Multifactorial interventions significantly reduced the total number of falls but did not have a significant effect on the total number of fallers or recurrent falls. The study highlights the need for further research on the effectiveness of exercise in individuals with cognitive impairment and dementia, as these groups are often excluded from research.
[24] (2020) Canada	Quality Improvement Study<br><br>To evaluate the impact of implementing the Fall Tailoring Interventions for Patient Safety (TIPS) program on fall and fall-related injury prevention in older nursing home residents in Canada.<br><br>Participants: 15 residents	Assessment, Environmental, Education	Fall Risk Assessment:<br>Individualized assessment of risk factors using validated scales such as the Morse Fall Scale. Continuous monitoring and regular review of the prevention plan to ensure appropriateness.<br>Exercise Programs:<br>No specific description of physical exercises in the Fall TIPS intervention; the focus was primarily on assessment and planning.<br>Environmental Modifications:<br>Personalized environmental adjustments as part of the individualized prevention plan, including use of walking aids and additional supervision.

			Educational Programs:<br>Active education and involvement of residents and their families in the prevention process. Ongoing staff training and education, though challenged by high staff turnover. Use of personalized posters to facilitate understanding of recommendations. Empowerment of residents to adhere to preventive strategies.<br>Results:<br>• Reduction in the average fall rate from 10.07 to 7.95 falls per 1,000 resident-days<br>• Reduction in the average rate of falls with injury from 2.91 to 1.01 falls with injury per 1,000 resident-days
[25] (2022) China	Quasi-Experimental, Pre-Test and Post-Test Study with Two Groups<br><br>To evaluate the feasibility and impact of the group-based Otago Exercise Program (OEP) on fear of falling (FOF) and physical function in older adults residing in nursing homes in China.<br>Sample: 57 participants (29 intervention group, 28 control group)<br>Inclusion criteria:<br>• Age ≥ 65 years<br>• Ability to walk unaided (or with a cane)<br>• Residency in the institution for ≥ 3 months<br>• Independence in activities of daily living (BI ≥ 60)<br>Exclusion criteria:<br>• Severe cognitive impairment (MMSE < 10)<br>• Blindness or deafness<br>• Unstable medical conditions, stroke, epilepsy, among others	Assessment, Exercise, Education	Fall Risk Assessment:<br>• Fear of falling: mSAFFE scale<br>• Balance: Four-Stage Balance Test (FSBT)<br>• Mobility: Timed Up and Go test (TUG)<br>• Lower limb muscle strength: 30-Second Sit-to-Stand Test (30s-SST)<br>Exercise Programs:<br>Duration: 12 weeks, group-based Otago Exercise Program sessions (minimum 2x/week, 40–60 minutes) + walking (minimum 2x/week)<br>• Otago Program Components:<br>o Warm-up: marching in place, knee lifts, ankle mobilization, shoulder rotations, gentle stretching<br>o Strength training: 5 lower limb exercises (e.g., sit-to-stand without support, heel and toe raises, knee extension and flexion)<br>o Balance training: 12 progressive exercises, including single-leg stance, tandem walking, squats, step-ups, and more<br>o Walking: 30 minutes on days alternating with exercise sessions<br>Educational Programs:<br>Illustrated Otago program manual provided to participants<br>Adherence incentives via coupons exchangeable for small gifts

			Reminders sent 20 minutes before sessions to improve participation<br>Results:<br>• Intervention group showed significant improvement in all measures<br>• Control group showed deterioration in TUG, FSBT, and 30s-SST
[26]<br>(2020)<br>Portugal	Integrative Literature Review<br><br>The study aimed to identify the instruments used to assess fall risk in institutionalized older adults, addressing the research question: "Which assessment instruments are used to determine fall risk in institutionalized older adults?"	Assessment	Fall Risk Assessment:<br>Functional tests: Timed Up and Go Test (TUGT), Performance-Oriented Mobility Assessment (POMA), Berg Balance Test (BBS)<br>Specific scales: Morse Falls Scale (MFS), Downton Fall Risk Index, Hendrich Fall Risk Model-II (HFRM-II), among others<br>Cognitive assessment: Mini-Mental State Examination (MMSE) to identify cognitive deficits associated with fall risk<br>Key question: "Have you fallen in the past 12 months?" – strong predictive value for future falls<br>Results:<br>• No scale was specifically developed for institutionalized older adults<br>• Combined use of multiple instruments may be impractical due to time constraints<br>• Initial assessment should be performed within the first 24–48 hours after admission<br>• Lack of sensitivity and specificity in many instruments may lead to underestimation or overestimation of fall risk
[27]<br>(2020)<br>Canada	Retrospective Cohort Study<br><br>To evaluate the predictive accuracy of the interRAI Falls Clinical Assessment Protocol (CAP), the Scott Fall Risk Screen (SFRS), and an additional fall risk assessment tool used in long-term residential care.<br><br>Participants: 1,553 residents	Assessment	Fall Risk Assessment:<br>• interRAI Falls CAP: best overall accuracy; c-statistic 0.673, specificity 0.834, sensitivity 0.502<br>• Supplementary FRA: very high sensitivity 0.943 but low specificity 0.111<br>• Scott Fall Risk Screen (SFRS): intermediate results<br>The inclusion of clinical diagnoses (Parkinson's disease, Alzheimer's disease, COPD,

			cardiovascular diseases) improved the accuracy of the tools, particularly the interRAI CAP (c-statistic increased to 0.749).
[28] (2021)<br><br>Turkey	Randomized Controlled Trial<br>To evaluate the effects of the Otago Exercise Program (OEP) on fall reduction, balance improvement, and physical performance in high fall-risk older adults residing in a nursing home in Turkey.<br>Sample: 71 participants (35 in the Otago group, 36 in the walking group)<br>Inclusion criteria:<br>• Age ≥ 65 years<br>• Ability to read and write in Turkish<br>• Independence in activities of daily living (Katz Index ≥ 5)<br>• High fall risk (Itaki Fall Risk Scale ≥ 5)<br>Exclusion criteria:<br>• Palliative care<br>• Severe sensory deficits<br>• Dementia<br>• Hypotension<br>• Anemia<br>• Decompensated metabolic/acute diseases<br>• Arrhythmia<br>• Uncontrolled hypertension<br>• Angina<br>• Severe venous insufficiency<br>• Recent surgery (<6 weeks)<br>• Physical incapacity to perform exercises	Exercise, Education	Exercise Programs:<br>Supervised training 3x/week for 1 month, followed by self-directed supervised training with weekly follow-up during months 2 and 3. Exercises included warm-up, lower limb strength training (sit-to-stand, heel/toe raises, knee flexion/extension), and balance training (feet together, single-leg stance, obstacle walking, lateral transfers, reaching movements). Supervised walking alternated with strength training days.<br><br>Educational Programs:<br>Use of visual materials (projector) and illustrated pamphlets to facilitate exercise learning, supporting participant adherence and understanding.<br><br>Results:<br>The Otago group showed a significant reduction in the number of falls, improved balance (Berg Balance Scale +6 points), and increased lower limb strength (+4 repetitions on the 30s Chair Stand Test). The walking group showed no significant changes.

PRISMA 2020 Checklist

Section and Topic	Item #	Checklist item	Location where item is reported
**TITLE**			
Title	1	Identify the report as a systematic review.	yes
**ABSTRACT**			
Abstract	2	See the PRISMA 2020 for Abstracts checklist.	Followed
**INTRODUCTION**			
Rationale	3	Describe the rationale for the review in the context of existing knowledge.	Line 103-110
Objectives	4	Provide an explicit statement of the objective(s) or question(s) the review addresses.	Line 112-120
**METHODS**			
Eligibility criteria	5	Specify the inclusion and exclusion criteria for the review and how studies were grouped for the syntheses.	Line 150-151
Information sources	6	Specify all databases, registers, websites, organisations, reference lists and other sources searched or consulted to identify studies. Specify the date when each source was last searched or consulted.	Line 99-102<br>Line 112-115
Search strategy	7	Present the full search strategies for all databases, registers and websites, including any filters and limits used.	Table 2
Selection process	8	Specify the methods used to decide whether a study met the inclusion criteria of the review, including how many reviewers screened each record and each report retrieved, whether they worked independently, and if applicable, details of automation tools used in the process.	Line 190-195
Data collection process	9	Specify the methods used to collect data from reports, including how many reviewers collected data from each report, whether they worked independently, any processes for obtaining or confirming data from study investigators, and if applicable, details of automation tools used in the process.	Line 198-204
Data items	10a	List and define all outcomes for which data were sought. Specify whether all results that were compatible with each outcome domain in each study were sought (e.g. for all measures, time points, analyses), and if not, the methods used to decide which results to collect.	Line 131-134
	10b	List and define all other variables for which data were sought (e.g. participant and intervention characteristics, funding sources). Describe any assumptions made about any missing or unclear information.	Line 131-134
Study risk of bias assessment	11	Specify the methods used to assess risk of bias in the included studies, including details of the tool(s) used, how many reviewers assessed each study and whether they worked independently, and if applicable, details of automation tools used in the process.	Line 206-210
Effect measures	12	Specify for each outcome the effect measure(s) (e.g. risk ratio, mean difference) used in the synthesis or presentation of results.	N/A
Synthesis methods	13a	Describe the processes used to decide which studies were eligible for each synthesis (e.g. tabulating the study intervention characteristics and comparing against the planned groups for each synthesis (item #5)).	N/A
	13b	Describe any methods required to prepare the data for presentation or synthesis, such as handling of missing summary statistics, or data conversions.	N/A
	13c	Describe any methods used to tabulate or visually display results of individual studies and syntheses.	N/A
	13d	Describe any methods used to synthesize results and provide a rationale for the choice(s). If meta-analysis was performed, describe the model(s), method(s) to identify the presence and extent of statistical heterogeneity, and software package(s) used.	N/A
	13e	Describe any methods used to explore possible causes of heterogeneity among study results (e.g. subgroup analysis, meta-regression).	N/A
	13f	Describe any sensitivity analyses conducted to assess robustness of the synthesized results.	N/A

PRISMA 2020 Checklist

Section and Topic	Item #	Checklist item	Location where item is reported
Reporting bias assessment	14	Describe any methods used to assess risk of bias due to missing results in a synthesis (arising from reporting biases).	N/A
Certainty assessment	15	Describe any methods used to assess certainty (or confidence) in the body of evidence for an outcome.	N/A
**RESULTS**			
Study selection	16a	Describe the results of the search and selection process, from the number of records identified in the search to the number of studies included in the review, ideally using a flow diagram.	Fig.1, page 7
	16b	Cite studies that might appear to meet the inclusion criteria, but which were excluded, and explain why they were excluded.	Fig.1, page 7
Study characteristics	17	Cite each included study and present its characteristics.	Line 239-258
Risk of bias in studies	18	Present assessments of risk of bias for each included study.	N/A
Results of individual studies	19	For all outcomes, present, for each study: (a) summary statistics for each group (where appropriate) and (b) an effect estimate and its precision (e.g. confidence/credible interval), ideally using structured tables or plots.	Table 3, page 8-10
Results of syntheses	20a	For each synthesis, briefly summarise the characteristics and risk of bias among contributing studies.	N/A
	20b	Present results of all statistical syntheses conducted. If meta-analysis was done, present for each the summary estimate and its precision (e.g. confidence/credible interval) and measures of statistical heterogeneity. If comparing groups, describe the direction of the effect.	N/A
	20c	Present results of all investigations of possible causes of heterogeneity among study results.	N/A
	20d	Present results of all sensitivity analyses conducted to assess the robustness of the synthesized results.	N/A
Reporting biases	21	Present assessments of risk of bias due to missing results (arising from reporting biases) for each synthesis assessed.	N/A
Certainty of evidence	22	Present assessments of certainty (or confidence) in the body of evidence for each outcome assessed.	N/A
**DISCUSSION**			
Discussion	23a	Provide a general interpretation of the results in the context of other evidence.	Line 318-393
	23b	Discuss any limitations of the evidence included in the review.	Line 404-415
	23c	Discuss any limitations of the review processes used.	Line 404-415
	23d	Discuss implications of the results for practice, policy, and future research.	Line 394-403
**OTHER INFORMATION**			
Registration and protocol	24a	Provide registration information for the review, including register name and registration number, or state that the review was not registered.	Line 129-130
	24b	Indicate where the review protocol can be accessed, or state that a protocol was not prepared.	Line 129
	24c	Describe and explain any amendments to information provided at registration or in the protocol.	N/A

PRISMA 2020 Checklist

Section and Topic	Item #	Checklist item	Location where item is reported
Support	25	Describe sources of financial or non-financial support for the review, and the role of the funders or sponsors in the review.	Line 450-452
Competing interests	26	Declare any competing interests of review authors.	Line 455-456
Availability of data, code and other materials	27	Report which of the following are publicly available and where they can be found: template data collection forms; data extracted from included studies; data used for all analyses; analytic code; any other materials used in the review.	Line 453-454

From: Page MJ, McKenzie JE, Bossuyt PM, Boutron I, Hoffmann TC, Mulrow CD, et al. The PRISMA 2020 statement: an updated guideline for reporting systematic reviews. BMJ 2021;372:n71. doi: 10.1136/bmj.n71.
This work is licensed under CC BY 4.0. To view a copy of this license, visit https://creativecommons.org/licenses/by/4.0/
